# Adaptation to Photooxidative Stress: Common and Special Strategies of the Alphaproteobacteria *Rhodobacter sphaeroides* and *Rhodobacter capsulatus*

**DOI:** 10.3390/microorganisms8020283

**Published:** 2020-02-19

**Authors:** Mathieu K. Licht, Aaron M. Nuss, Marcel Volk, Anne Konzer, Michael Beckstette, Bork A. Berghoff, Gabriele Klug

**Affiliations:** 1 Institute of Microbiology and Molecular Biology, University of Giessen, 35392 Giessen, Germany; Mathieu.Licht@mikro.bio.uni-giessen.de (M.K.L.); Marcel.Volk@ukmuenster.de (M.V.); 2 Department of Molecular Infection Biology, Helmholtz Centre for Infection Research, 38124 Braunschweig, Germany; aaron.nuss@microsynth.seqlab.de; 3 Institute for Infectiology, Center for Molecular Biology of Inflammation, University of Münster, 48149 Münster, Germany; 4 Biomolecular Mass Spectrometry, Max Planck Institute for Heart and Lung Research, 61231 Bad Nauheim, Germany; anne.konzer@gmail.com; 5 Department of Computational Biology for Individualized Medicine, Centre for Individualized Infection Medicine, 30625 Hannover, Germany; Michael.Beckstette@helmholtz-hzi.de

**Keywords:** *Rhodobacter capsulatus*, *Rhodobacter sphaeroides*, photooxidative stress, transcriptomics, proteomics, stress defense

## Abstract

Photosynthetic bacteria have to deal with the risk of photooxidative stress that occurs in presence of light and oxygen due to the photosensitizing activity of (bacterio-) chlorophylls. Facultative phototrophs of the genus *Rhodobacter* adapt the formation of photosynthetic complexes to oxygen and light conditions, but cannot completely avoid this stress if environmental conditions suddenly change. *R. capsulatus* has a stronger pigmentation and faster switches to phototrophic growth than *R. sphaeroides*. However, its photooxidative stress response has not been investigated. Here, we compare both species by transcriptomics and proteomics, revealing that proteins involved in oxidation–reduction processes, DNA, and protein damage repair play pivotal roles. These functions are likely universal to many phototrophs. Furthermore, the alternative sigma factors RpoE and RpoH_II_ are induced in both species, even though the genetic localization of the *rpoE* gene, the RpoE protein itself, and probably its regulon, are different. Despite sharing the same habitats, our findings also suggest individual strategies. The *crtIB-tspO* operon, encoding proteins for biosynthesis of carotenoid precursors and a regulator of photosynthesis, and *cbiX*, encoding a putative ferrochelatase, are induced in *R. capsulatus*. This specific response might support adaptation by maintaining high carotenoid-to-bacteriochlorophyll ratios and preventing the accumulation of porphyrin-derived photosensitizers.

## 1. Introduction

Microbes in aquatic habitats need to adapt to frequent changes in environmental parameters like temperature, O_2_-saturation, or light conditions. While phototrophic bacteria can take advantage of pigment-protein complexes to use light energy for ATP production, they face the special challenge of photooxidative stress: (bacterio-) chlorophylls can act as photosensitizers and transfer energy to the ground state triplet oxygen (^3^O_2_), causing a spin conversion in the π*2p orbital that generates highly reactive singlet oxygen (^1^O_2_). While other photosensitizers like humic acids also contribute to photooxidative stress, (bacterio-) chlorophyll *a* is regarded as the main cause of ^1^O_2_-generation in photosynthetic bacteria. Independently of light, processes like lipid peroxide decomposition or hypochloric acid reacting with hydrogen peroxide can generate ^1^O_2_ [1].

Facultative anoxygenic phototrophic bacteria of the genus *Rhodobacter* adjust their lifestyle to the light and oxygen conditions. Due to a high metabolic versatility, they do not rely on photosynthesis for ATP production, but can also perform aerobic or anaerobic respiration or fermentation. They do not form photosynthetic complexes at high oxygen concentrations, and at intermediate oxygen concentration, light inhibits the accumulation of pigment-protein complexes [2,3], which reduces the risk of photooxidative stress. Several protein regulators, including redox-responsive factors, photoreceptors, and even proteins with dual-sensing function like AppA [2], but also RNA regulators [4,5], contribute to the regulated formation of photosynthetic complexes.

Nevertheless, situations that cause photooxidative stress cannot be completely avoided, and consequently, mechanisms to defend this stress are important for survival. As seen across all kingdoms, ^1^O_2_ damages a wide variety of biomolecules, including nucleic acids, amino acids, fatty acid lipids or thiols, and glutathione [1,6,7]. Singlet oxygen can directly oxidize its targets or generate other reactive oxygen species (ROS) like endo- or hydroperoxides via (4 + 2) cycloaddition or the ene reaction [8]. Without a proper cellular response, ^1^O_2_-stress can be cytotoxic [9,10]. In the case of DNA, the mutagenic potential of ^1^O_2_ in *Escherichia coli* can be assigned to the oxidation of guanine sites to 8-oxo-7,8-dihydro-2′-deoxyguanosine (8-OHdG), which is susceptible to single strand breaks [9,11]. Regardless of the occurrence of 8-OHdG, ^1^O_2_ can also affect RNA, specifically viral RNA, by mediating RNA-protein-crosslinking, as shown for photoinactivation of HIV-1 [12]. As ^1^O_2_ can form peroxides, it also targets unsaturated fatty acids, causing lipid peroxidation which impairs membranes in their potential, integrity, or transport activities [13,14]. However, due to their high abundance in the cell, proteins are the primary targets of ^1^O_2_ [15]. Protein damage by ^1^O_2_ may often be traced back to the oxidization of amino acids containing sulfur or aromatic compounds [7,16], but other ROS generated by ^1^O_2_ might target the proteome as well. Unfolded or aggregated proteins and the loss of enzyme activities are likely consequences of ^1^O_2_ [17].

For more than a decade, *R. sphaeroides* have served as the bacterial model organism to elucidate the photooxidative stress response. Increased expression of certain genes in response to ^1^O_2_ was demonstrated, and an important role of the alternative sigma factors RpoE, RpoH_I_, and RpoH_II_ in this response was revealed [10,18,19,20,21]. An early step in ^1^O_2_-dependent gene activation is proteolytic degradation of the antisigma factor ChrR [22,23]. The released RpoE sigma factor directly activates a small number of genes like the DNA photolyase gene *phrA* or *cfaS* (cyclopropane fatty acyl-phospholipid synthase) [24,25], but also the *rpoH_II_* gene. RpoH_II_, together with RpoH_I_, activates a high number of genes upon photooxidative stress, but also in response to other stresses [20,21]. Genes that are activated upon photooxidative stress have functions, e.g., in the detoxification of toxic molecules like peroxides or methylglyoxal, in protein quality control and turnover, in ^1^O_2_ quenching, DNA repair, and transport [1,26]. Although carotenoids provide protection against ^1^O_2_ in *R. sphaeroides*, genes for carotenoid synthesis are not activated by ^1^O_2_ in this bacterium [27,28].

Regarding the photoprotective function of carotenoids, both *R. capsulatus* and *R. sphaeroides* accumulate mainly spheroidene (SE) under anaerobic conditions and spheroidenone (SO) under (semi-) aerobic conditions [27,29,30,31,32,33]. An oxygen-activated spheroidene monooxygenase (CrtA) causes this shift and incorporates a keto-group into SE to form SO [31,32]. The shift from SE to SO helps *Rhodobacter* to counteract ^1^O_2_ [34]. The introduced keto-group stabilizes the intramolecular charge transfer state of excited carotenoids by binding to the reaction center of the light-harvesting complex I. Due to its low energy, the intramolecular charge transfer state of carotenoids enables the quenching of ^1^O_2_ without sacrificing light-harvest. This could explain why different carotenoid-deficient mutants of *R. sphaeroides* showed decreased survival rates under photooxidative stress when (hydroxy-) SO amounts were very low [27].

Many small RNAs (sRNAs) are induced by ^1^O_2_ [35], and for some, the regulatory function could be elucidated. The sRNA-mRNA interactions are often stabilized by the RNA chaperone Hfq, which is another crucial element of the photooxidative stress response in *R. sphaeroides* [36]. Some of these sRNAs (CcsR1-4, Pos19) are involved in balancing the glutathione pool and in the downregulation of the pyruvate dehydrogenase complex and aerobic electron transport, a primary source of ROS [37,38]. Other sRNAs (SorY, SorX) reduce the metabolic flux into the tricarboxylic acid (TCA) cycle or affect polyamine transport [39,40]. A switch from glycolysis to the pentose phosphate cycle and reduced activity of the TCA cycle upon oxidative stress reduce the production of the pro-oxidant NADH and increase production of the protective NADPH. An integrative “omics” approach supports the importance of posttranscriptional regulation in the ^1^O_2_ response of *R. sphaeroides* [28].

*R. capsulatus*, another member of the *Rhodobacteraceae*, shares a very similar life style with *R. sphaeroides*, and was also intensely studied with regard to its adaptation to different oxygen- and light conditions [41,42]. Under high oxygen tension, *R. capsulatus* cultures show more pigmentation than *R. sphaeroides*, implying a faster adaptation to phototrophic conditions but a higher risk of ^1^O_2_ production. However, the response to ^1^O_2_ has not been elucidated in *R. capsulatus*. In this study, we applied omics approaches to analyze and compare the response of the two *Rhodobacter* species to photooxidative stress. Although both species share the same habitats, our findings suggest individual strategies to defend against photooxidative stress in addition to a common core response.

## 2. Materials and Methods

### 2.1. Bacterial Strains and Growth Conditions

*Rhodobacter* strains (Table S1) were cultivated at 32 °C in minimal medium containing malate as a carbon source [43]. For microaerobic conditions (~25 µM O_2_), cultures were incubated in Erlenmeyer flasks with a culture volume of 80% and shaking at 140 rpm. To cultivate *Rhodobacter* under aerobic conditions (160–180 µM O_2_), cultures were grown either in baffled flasks with shaking at 140 rpm and a culture volume of 20%, or in flat glass bottles gassed with air. To establish phototrophic growth, airtight flat glass bottles were completely filled with medium and cultures were illuminated continuously with white light (60 W·m^−2^; fluorescent tube: Omnilux 18W). In order to shift *Rhodobacter* between two different growth conditions, exponentially growing cultures (OD_660_ of ~0.4) were diluted to an OD_660_ of 0.2.

### 2.2. Photooxidative Stress Experiments

Photooxidative stress experiments were carried out as previously described in Glaeser and Klug, 2005 [27]. In short, pigmented cultures from microaerobic cultivation were shifted to aerobic conditions in air-gassed flat glass bottles in the dark. Methylene blue was added at a final concentration of 0.2 µM. After an OD_660_ of ~0.4 was reached, cultures were exposed to 800 W·m^−2^ white light to generate ^1^O_2_ (photooxidative stress).

For zone of inhibition assays, exponentially growing cultures were diluted into soft agar (0.8%, *w*/*v*) and poured onto malate minimal salt medium agar (1.6%, *w*/*v*). Five microliters of methylene blue (10 µM) were spotted onto a filter paper disk, which was placed in the center of the agar plate. Cultures were incubated for 48 h at 32 °C under illumination with 20 W·m^−2^ white light.

### 2.3. Analysis of Pigmentation

BChl *a* and carotenoids were extracted and measured as described in Glaeser and Klug, 2005 [27]. Briefly, 1 mL samples of *Rhodobacter* cultures were harvested at 17,000× *g* for 5 min. Pellets were resuspended in 50 µL ddH_2_O and mixed with 500 µL of acetone/methanol (7/2, *v*/*v*) by vortexing for 30 s. Samples were centrifuged at 17,000× *g* for 5 min, and the absorption of the supernatant was measured in a Specord 50 Plus spectrometer (Analytik Jena, Jena, Germany), using acetone/methanol (7/2, *v*/*v*) as a reference. The carotenoid and BChl *a* concentrations were calculated from the absorptions at 484 and 770 nm, respectively, with extinction coefficients of 128 mM^−1^·cm^−1^ for carotenoids [44] and 76 mM^−1^·cm^−1^ for BChl *a* [45]. Concentrations were normalized to the OD_660_.

### 2.4. Measurement of Reactive Oxygen Species

Singlet oxygen levels were measured using the fluorescent probe Singlet Oxygen Sensor Green (SOSG, Molecular Probes, Eugene, OR, USA). A SOSG stock solution of 100 µM was prepared in HEPES buffer (40 mM, pH 7, 1% methanol). Six microliters of the SOSG stock solution were added to 114 µL culture samples (final SOSG concentration of 5 µM). Technical duplicates were incubated for 30 min at 32 °C and 450 rpm in a Vibramax 100 shaker (Heidolph Instruments, Schwabach, Germany). Samples were either kept in the dark or illuminated with 800 W·m^−2^ red light. Samples without SOSG served as background controls. Cells were centrifuged at 8,000 rpm for 5 min and resuspended in 100 µL HEPES buffer (40 mM, pH 7, 1% methanol). Fluorescence intensities (excitation 500 nm, emission 532 nm) were measured in an Infinite M200 microplate reader (Tecan, Crailsheim, Germany). After subtraction of the background control, fluorescence intensities were normalized to BChl *a* levels. Ratios between illuminated samples and dark controls were subsequently calculated.

General ROS levels were measured as previously described [43], using the oxidation-sensitive fluorescent probe 2,7-dihydrodichlorofluorescein diacetate (H_2_DCFDA, Molecular Probes, Eugene, OR, USA). Culture samples of 100 µL were incubated at 32 °C with H_2_DCFDA (final concentration of 10 µM) for 30 min and shaking at 140 rpm in technical triplicates. A culture sample without H_2_DCFDA served as background control. Fluorescence intensities (excitation 492 nm, emission 525 nm) were measured in an Infinite M200 microplate reader (Tecan, Crailsheim, Germany). After subtraction of the background control, fluorescence intensities were normalized to the OD_660_.

### 2.5. Transcriptome Analysis by RNA-Sequencing

#### 2.5.1. Sample Preparation for RNA-seq

Cultures of *R. capsulatus* were shifted from microaerobic to aerobic growth in the dark followed by photooxidative stress as described by Berghoff and colleagues [28]. Samples of 20 mL before (0 min) and after stress (10 min) were collected, cooled on ice, and centrifuged at 10,000× *g* for 10 min at 4 °C. Cell pellets were resuspended in 1 mL minimal medium and centrifuged at 10,000× *g* for 10 min at 4 °C. RNA was extracted via the hot phenol protocol [46]. The RNA was resolved in RNase-free water (Roth) and treated with DNaseI (Invitrogen, Carlsbad, CA, USA) to remove traces of DNA. A test PCR (rpoZ-for: 5′-GAT GAT CTG CGC GAG CGT CT-3′; rpoZ-rev: 5′-CCT TGC GCG TCC ATC AAT GC-3′) was performed to ensure that the RNA was free of DNA. RNA integrity was assessed using the Agilent RNA 6000 Nano Kit on the Agilent 2100 Bioanalyzer (Agilent Technologies, Santa clara, CA, USA) to ensure high quality RNA (RIN ≥ 9) for downstream processing. rRNA was depleted from 5 µg of total RNA using the Ribo-Zero rRNA Removal Kit (Gram-Negative Bacteria, Epicentre Biotechnologies, Madison, WI, USA) as recommended by the manufacturer. One microliter of either 1:10 diluted ERCC ExFold RNA Spike-in Mix 1 or Mix 2 (Ambion, Austin, TX, USA) was added to 1 µg of rRNA-depleted RNA. To create 5′-monophosphorylated RNA, rRNA-depleted RNA (including ERCC Spike-in Mixes) was treated with RNA 5′ polyphosphatase as recommended by the manufacturer.

#### 2.5.2. Strand-Specific Library Preparation and Illumina Sequencing

Strand-specific RNA-seq cDNA library preparation and barcode introduction was based on RNA adapter ligation as described earlier [47]. The quality of the libraries was validated using an Agilent 2100 Bioanalyzer (Agilent Technologies) following the manufacturer’s instruction. Cluster generation was performed using the Illumina cluster station. Single-end sequencing on the HiSeq2500 followed a standard protocol. The fluorescent images were processed to sequences and transformed to FastQ format using the Genome Analyzer Pipeline Analysis software 1.8.2 (Illumina, San Diego, CA, USA). The sequence output was controlled for general quality features, sequencing adapter clipping, and demultiplexing using the fastq-mcf and fastq-multx tool of ea-utils [48].

#### 2.5.3. Read Mapping, Bioinformatics and Statistics

The quality of the sequencing output and potential contamination was analyzed using FastQC (Babraham Bioinformatics, http://www.bioinformatics.babraham.ac.uk/projects/fastqc/). Identified adapter contamination and remaining artificial sequence (barcode) were removed using program fastx_trimmer from the FASTX-210 toolkit version 0.0.13 (http://hannonlab.cshl.edu/fastx_toolkit/). On the 3′-end, reads were trimmed if the per base Phred score fell short of 20. Trimmed reads with a remaining length < 20 nucleotides were discarded. All sequenced libraries were mapped to the *R. capsulatus* genome (accession no. NC_014034) and the pRCB133 plasmid (accession no. NC_014035.1) using Bowtie2 (version 2.1.0) in end-to-end alignment mode [49]. After read mapping, the resulting bam files were filtered for uniquely mapped reads using SAMtools (both strands) [50]. The determined uniquely mapped read counts served as inputs to DESeq2 [51] for the pairwise detection and quantification of differential gene expression. For DESeq2 parametrization, we used a beta prior and disabled Cook distance cut off filtering. All other parameters remained unchanged. In addition, RPKM (reads per kilobase max. transcript length per million mapped reads) values were computed for each library from the raw gene counts. The list of DESeq2 determined differentially expressed genes (DEGs) was filtered with a conservative absolute log2 fold change cutoff of at least 1 and a cutoff for a multiple testing corrected *p*-value of at most 0.05.

#### 2.5.4. ERCC Spike-in Control Analysis

To assess the platform dynamic range and the accuracy of fold-change responses, ERCC RNA Spike-in controls were used. Spike-in control sequences were added to the *R. capsulatus* reference genome/annotation prior to read alignment and read counts for Spike-in controls were determined, along with normal gene counts with program htseq-count. Further data analyses and the generation of dose- and fold-change response plots were performed as described by the manufacturer (Ambion, Carlsbad, CA, USA).

#### 2.5.5. RNA-seq Data Accessibility

The RNA-seq analysis can be found in Table S2. Raw RNA-seq data have been deposited in NCBI’s Gene Expression Omnibus, and are accessible through GEO Series accession number GSE134200.

### 2.6. Quantitative RT-PCR

For quantitative RT-PCR, total RNA was isolated after 10 min of photooxidative stress as described for RNA-seq. Samples were treated with TURBO DNA-free™ Kit (Invitrogen, Thermo Fisher Scientific, Schwerte, Germany) to remove DNA contaminations. The Brilliant III Ultra-Fast SYBR Green QRT-PCR Master Mix (Agilent Technologies, Waldbronn, Germany) was applied using 4 ng·µL^−1^ of total RNA per reaction. RT-PCR was performed in a CFX Connect™ Real-Time System (Bio-Rad). Cycle threshold (Ct) values were determined using the CFX Maestro™ Software (Bio-Rad, Feldkirchen, Germany), and relative transcript levels calculated according to Pfaffl (2001) [52]. The *rpoZ* gene was used for normalization. Primers and their amplification efficiencies are listed in Table S3.

### 2.7. Protein Sample Preparation and Mass Spectrometry

Protein sample preparation was performed as previously described [53]. For mass spectrometry (MS) analysis, peptides were eluted from STAGE tips by solvent B (80% acetonitrile, 0.1% formic acid), dried down in a SpeedVac Concentrator (Thermo Fisher Scientific, Schwerte, Germany) and dissolved in solvent A (0.1% formic acid). Peptides were separated using an UHPLC system (EASY-nLC 1000, ThermoFisher Scientific, Waltham, MA, USA) and 20 cm, in-house packed C18 silica columns (1.9 µm C18 beads, Dr. Maisch GmbH) coupled in line to a Q-Exactive HF orbitrap mass spectrometer (ThermoFisher Scientific) using an electrospray ionization source. A gradient of 240 min was applied using a linearly increasing concentration of solvent B (80% acetonitrile, 0.1% formic acid) over solvent A (0.1% formic acid) from 5% to 30% for 215 min and from 30% to 60% for 5 min, followed by washing with 95% of solvent B for 5 min and re-equilibration with 5% of solvent B. Full MS spectra were acquired in a mass range of 300 to 1750 m/z with a resolution of 60,000 at 200 m/z. The ion injection target was set to 3 × 10^6^ and the maximum injection time limited to 20 ms. Ions were fragmented by high-energy collision dissociation (HCD) using a normalized collision energy of 27 and an ion injection target of 5 × 10^5^ with a maximum injection time of 20 ms. The resulting tandem mass spectra (MS/MS) were acquired with a resolution of 15,000 at 200 m/z using data dependent mode with a loop count of 15 (top 15). MS raw data were processed by MaxQuant (1.5.3.12) [54] using the Uniprot database for *R. capsulatus* containing 4290 entries (release date July 2016). The following parameters were used for data processing: maximum of two miss cleavages, mass tolerance of 4.5 ppm for main search, trypsin as digesting enzyme, carbamidomethylation of cysteines as fixed modification, oxidation of methionine, and acetylation of the protein N-terminus as variable modifications. For protein quantification, the LFQ function of MaxQuant was used. Peptides with a minimum of seven amino acids and at least one unique peptide were required for protein identification. Only proteins with at least two peptides and at least one unique peptide were considered to have been identified and were used for further data analysis. LFQ intensities for all identified proteins can be found in Table S4.

### 2.8. Search for Orthologous Rhodobacter Genes and Synteny Analysis

To find orthologous genes in *R. capsulatus* and *R. sphaeroides,* the Genome Gene Best Homologs tool from the IMG web resources was used [55]. The pBLAST-based search of orthologous genes used a 30% amino acid identity as a cutoff value for homology. Phyre^2^ was applied for a structural homology search [56]. Synteny analysis was performed using Edgar 2.3, a software platform for comparative gene content analyses [57].

### 2.9. Gene Ontology Enrichment Analysis

Significantly enriched functional groups were determined with the program Cytoscape version 3.6.0 [58] according to Gene Ontology (GO) terms using the BiNGO tool [59]. Overrepresented GO categories in the data sets were determined with a hypergeometric test with Benjamini-Hochberg false discovery rate correction and a significance level of 0.05. The whole *R. capsulatus* genome served as a reference. The selected ontology file was *gb.obo*, format-version 1.2, released 06/10/2017 [60,61]. The resulting networks were searched for overrepresented GO categories.

## 3. Results

### 3.1. Adaptation of R. sphaeroides and R. capsulatus to Different Growth Conditions

**Figure 1:**
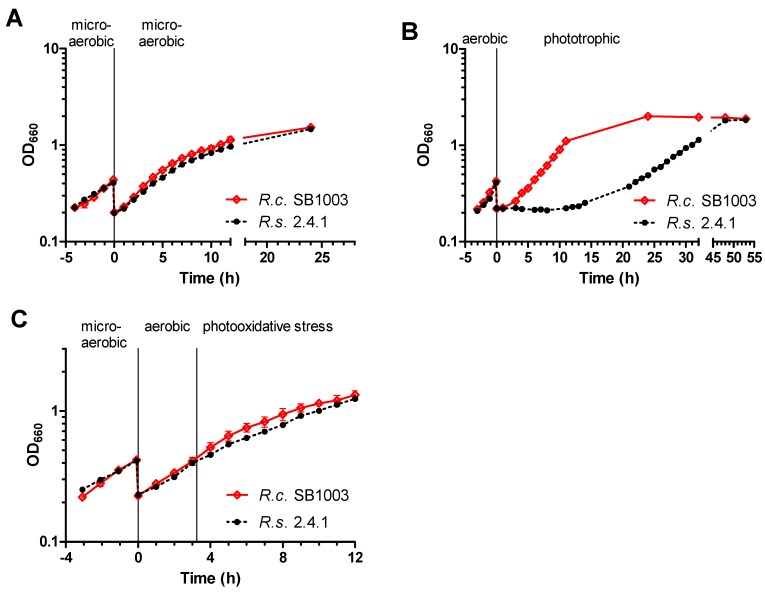
Growth of *R. capsulatus* and *R. sphaeroides* under different oxygen and light conditions. Exponential phase cultures of *R. capsulatus* (*R.c.* SB1003) and *R. sphaeroides* (*R.s.* 2.4.1) were diluted to an OD_660_ of 0.2 at time point 0 h. The OD_660_ was plotted semi-logarithmically against the time. Data points represent the mean of biological triplicates and error bars depict the standard deviation (standard deviations might not be visible if they are too small). (**A**) Microaerobically growing cultures. (**B**) Aerobically growing cultures shifted to phototrophic growth. (**C**) Microaerobically growing cultures were exposed to photooxidative stress when an OD_660_ of ~0.4 was reached. Figure S2 shows growth under dark and light conditions with or without methylene blue.

Based on the different pigment content of the two *Rhodobacter* species, we hypothesized differences in their adaptation to phototrophic growth. When cultures of the two species were kept under microaerobic conditions in the dark, the growth behavior was nearly identical (doubling time t_d_ of 4 h ± 3 min for *R. capsulatus* and 4 h 10 min ± 15 min for *R. sphaeroides*, Figure 1A). A shift from high oxygen to phototrophic conditions with 60 W·m^−2^ white light revealed a remarkably faster adaption process for *R. capsulatus*, i.e., entering the exponential growth after the shift took ~4 h for *R. capsulatus* but ~21 h for *R. sphaeroides* (Figure 1B). As seen by the doubling time, *R. capsulatus* also grew faster in exponential phase under phototrophic conditions than *R. sphaeroides* (t_d_ of 3 h 35 min ± 4 min for *R. capsulatus* and 6 h 50 min ± 30 min for *R. sphaeroides*). This supported our hypothesis that the higher pigment content of *R. capsulatus* would allow a faster switch to occur to phototrophic growth. Interestingly, carotenoids (especially SE and SO) strongly contributed to the growth benefit of *R. capsulatus*, as shown by experiments with transposon mutants lacking these carotenoids (Figure S1).

We also asked the question of how a strong increase of photooxidative stress caused by ^1^O_2_ would affect growth of the two *Rhodobacter* species. To test this, pigmented cultures (after microaerobic cultivation) were cultivated under aerobic conditions in the dark and shifted to high light conditions (800 W·m^−2^) in the presence of methylene blue (0.2 µM) when an OD_660_ of 0.4 was reached. These conditions were previously shown to produce ^1^O_2_ and to induce a specific response in *R. sphaeroides* [27,28]. Importantly, neither methylene blue in the dark nor high light without methylene blue resulted in a strong growth retardation (Figure S2). After initiating photooxidative stress, *R. capsulatus* showed faster growth than *R. sphaeroides*, but slowed down earlier. As a consequence, both strains reached the same OD_660_ after 12 h (Figure 1C).

**Figure 2:**
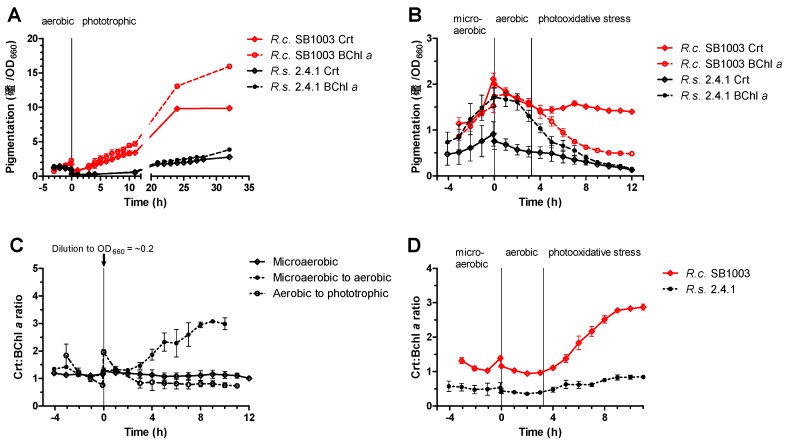
*R. capsulatus* has a stronger pigmentation and a higher carotenoid to bacteriochlorophyll *a* ratio than *R. sphaeroides*. Cultures in the exponential phase were diluted to an OD_660_ of 0.2 at time point 0 h. The content of carotenoids (Crt) and bacteriochlorophyll *a* (Bchl *a*) was normalized to the respective OD_660_-values and plotted against the time for (**A**) aerobically growing cultures shifted to phototrophic growth, and (**B**) microaerobically growing cultures shifted to aerobic dark conditions followed by exposure to photooxidative stress. The Crt:Bchl *a* ratio was plotted against the time for (**C**) *R. capsulatus* either continuously grown under microaerobic conditions or shifted to different oxygen and light conditions, and for (**D**) *R. capsulatus* and *R. sphaeroides* after a shift from microaerobic to aerobic dark conditions followed by photooxidative stress. Data points represent the mean of biological triplicates and error bars depict the standard deviation (standard deviations might not be visible if they are too small).

Although a stronger pigmentation of *R. capsulatus* cultures under high oxygen tension compared to *R. sphaeroides* was obvious, we wanted to quantify the differences and also analyze the ratio of carotenoids and bacteriochlorophylls throughout growth at different conditions. While bacteriochlorophyll functions as a photosensitizer that promotes the production of ^1^O_2_, carotenoids can quench ^1^O_2_, and are thus part of the defense system against ^1^O_2_. In general, *R. capsulatus* showed a higher amount of bacteriochlorophyll *a* (Bchl *a*) and carotenoids, e.g., under microaerobic conditions (Figure S3) at an OD_660_ of ~0.4: *R. capsulatus* had ~3.0 µM per OD_660_ carotenoids and ~2.8 µM per OD_660_ Bchl *a*, whereas *R. sphaeroides* had ~0.7 µM per OD_660_ carotenoids and ~2.0 µM per OD_660_ Bchl *a*. After a shift to phototrophic growth, carotenoid and Bchl *a* levels steadily increased in *R. capsulatus,* reaching levels that were much higher than under aerobic conditions (Figure 2A). Since *R. sphaeroides* stopped growing after this transition for nearly 13 h, the carotenoid and Bchl *a* levels stayed low during this time period and increased only slowly. Thirty-two hours after the shift, carotenoid and Bchl *a* levels were about 3.6-fold and 4.1-fold higher, respectively, in *R. capsulatus* (Figure 2A). After a shift from low to high oxygen tension and addition of methylene blue in the dark, the pigment level steadily dropped in both strains (Figure 2B). When illumination was started to generate ^1^O_2_, the Bchl *a* level dropped further. The carotenoid level in *R. capsulatus*, however, remained fairly constant (~1.4 µM per OD_660_), with a small peak (~1.6 µM per OD_660_) after four hours of stress. By contrast, *R. sphaeroides* showed a steady decline of carotenoids.

Not only the total amount of pigments may be important for adaptation of *Rhodobacter* species to changing conditions, but also the ratio of the ^1^O_2_-quenching carotenoids (Crt) to the ^1^O_2_-producing BChl *a*. In *R. capsulatus*, the Crt:BChl *a* ratio did not change much during continuous cultivation under microaerobic conditions (ratio of ~1.1) or after a shift from aerobic to phototrophic conditions (ratio of ~0.8; Figure 2C). However, a shift from microaerobic to aerobic conditions resulted in a strong increase of the Crt:Bchl *a* ratio from ~1.2 to ~3.0 in *R. capsulatus* (Figure 2C). Figure 2D compares the change in the Crt:Bchl *a* ratio between *R. sphaeroides* and *R. capsulatus* upon exposure to ^1^O_2_. The ratios remained fairly constant in both species during microaerobic growth and also after the shift to aerobic dark conditions in the presence of methylene blue. The ratios were ~1.1 in *R. capsulatus* and ~0.5 in *R. sphaeroides*. After the start of illumination and the production of ^1^O_2_, the Crt:Bchl *a* ratio increased in *R. capsulatus* from ~1.1 to nearly 3.0. In *R. sphaeroides* this ratio was below one under all conditions, and only increased from ~0.5 to ~0.8 after the initiation of photooxidative stress.

### 3.2. Generation of ROS in R. sphaeroides and R. capsulatus upon Photooxidative Stress

**Figure 3:**
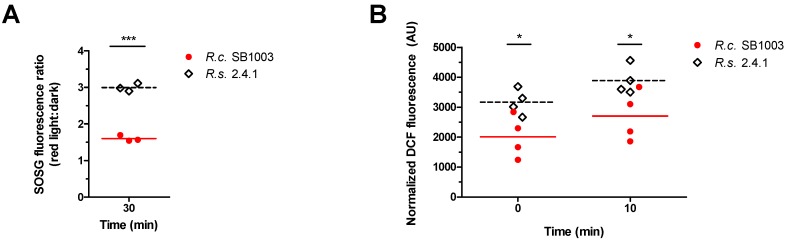
Determination of intracellular ^1^O_2_ and ROS levels in *R. capsulatus* and *R. sphaeroides* under photooxidative stress conditions. (**A**) The fluorogenic probe SOSG was used for ^1^O_2_ detection. Fluorescence intensities were normalized to BChl *a* levels. Ratios between illuminated samples (800 W·m^−2^ red light) and dark controls were calculated. (**B**) The fluorogenic probe H_2_DCFDA was used for ROS detection. Fluorescence intensities were normalized to the OD_660_ and displayed in arbitrary units (AU). Data points indicate individual measurements and bars represent the mean. Two-way ANOVA followed by Bonferroni posttest was used to compare results from *R. capsulatus* (*R.c.* SB1003) and *R. sphaeroides* (*R.s.* 2.4.1) (* *p*-value < 0.05, *** *p*-value <0.001).

To see whether the different Crt:BChl *a* ratios observed in the two *Rhodobacter* species under photooxidative stress conditions (Figure 2D) would affect ^1^O_2_ levels, the fluorescent probe Singlet Oxygen Sensor Green (SOSG) was used for in vivo ^1^O_2_ measurements. Since white light itself can affect SOSG fluorescence, red light was used for illumination [62]. In cell-free reactions, the combination of oxygen, red light, and methylene blue caused enhanced SOSG fluorescence due to photosensitized formation of ^1^O_2_ (Figure S4). When pigmented cultures were incubated under aerobic conditions in the presence of methylene blue, the ratio of SOSG fluorescence between illuminated samples and dark controls was significantly higher for *R. sphaeroides* than for *R. capsulatus* (Figure 3A), indicating enhanced ^1^O_2_ levels in *R. sphaeroides*. In addition, general ROS formation was measured by applying 2,7-dihydrodichlorofluorescein diacetate (H_2_DCFDA) before and after starting the white light illumination of aerobic cultures in the presence of methylene blue. The fluorogenic probe H_2_DCFDA is mainly specific for hydrogen peroxide, peroxynitrite anions, and peroxyl radicals [63], ROS that are partly generated downstream of ^1^O_2_ [15,64]. An increase in DCF fluorescence indicates elevated ROS levels. We found significant differences in fluorescence levels between the two species before and 10 min after initiating photooxidative stress (Figure 3B). The relative fluorescence intensity indicated higher ROS levels in *R. sphaeroides* compared to *R. capsulatus* by a factor of 1.6 and 1.5 for 0 and 10 min of photooxidative stress, respectively (Figure 3B).

### 3.3. The rpoE-chrR Locus of R. capsulatus Shows a Unique Genetic Context in Comparison to Other Bacteria within the Rhodobacteraceae

**Figure 4:**
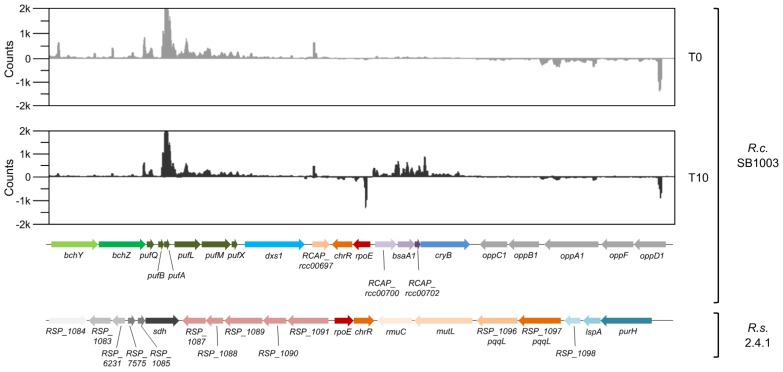
Microsynteny analysis of the *rpoE-chrR* locus in *R. capsulatus* and *R. sphaeroides*. The two upper panels show normalized read count distributions of a representative RNA-seq experiment with *R. capsulatus* (*R.c.* SB1003) before (T0) and 10 min after the onset of photooxidative stress (T10). The lower panels show the microsynteny analysis of the *rpoE-chrR* locus in *R. capsulatus* and its homologous genes in *R. sphaeroides* (*R.s.* 2.4.1). Homologs are indicated by identical colors.

Detailed work in *R. sphaeroides* identified the sigma factor RpoE as the master regulator of the response to ^1^O_2_ [1,65]. RpoE is primarily controlled by its cognate antisigma factor ChrR, but full activation of RpoE requires the RpoE regulon members RSP_1090/91 (putative cyclopropane/cyclopropene fatty acid synthesis proteins) and the cyclopropane-fatty-acyl-phospholipid synthase CfaS [22,23]. In addition, DegS and RseP homologous proteases are involved in the degradation of ChrR [22]. In *R. sphaeroides*, the RSP_1090/91 genes are located immediately upstream of the *rpoE-chrR* locus, a genetic arrangement that is conserved among many bacteria within the *Rhodobacteraceae* (e.g., *Roseobacter denitrificans*, *Dinoroseobacter shibae*, *Jannaschia rubra*, *Ruegeria litorea*, and *Oceanicola litoreus*). Interestingly, RpoE and ChrR homologs were not found in *R. capsulatus* using simple sequence alignment tools, but were revealed here by a structural homology search using Phyre^2^ [56]. The RpoE proteins of *R. sphaeroides* and *R. capsulatus* only share 24% identity, but the confidence in the Phyre^2^ structural homology analysis is high (99.9%). Importantly, the gene adjacent to the *rpoE* gene of *R. capsulatus* encodes a putative antisigma factor with 13% identity to ChrR (confidence of 99.4%). Although the identity values are relatively low, the high confidence in the Phyre^2^ analysis strongly suggests that *R. capsulatus* has true RpoE (RCAP_rcc00699) and ChrR (RCAP_rcc00698) homologs. Moreover, and similar to *R. sphaeroides*, the *rpoE-chrR* locus of *R. capsulatus* is induced by ^1^O_2_, as revealed by RNA-seq (Figure 4 and Table 1). However, microsynteny analysis using Edgar 2.3 [57] showed that the genetic context of the *rpoE-chrR* locus in *R. capsulatus* is different from *R. sphaeroides* (Figure 4). The RpoE-dependent operon RSP_1087-1091, which is located upstream of *rpoE-chrR* in *R. sphaeroides*, cannot be found next to *rpoE-chrR* in *R. capsulatus*. Moreover, respective homologs seem to be completely absent from the *R. capsulatus* genome (Table 1). Instead, the *rpoE-chrR* locus is located next to an operon, which encodes the glutathione peroxidase BsaA1, the cryptochrome/photolyase CryB, and two hypothetical proteins. All four genes are clearly induced by ^1^O_2_ (Figure 4). Interestingly, *cryB* belongs to the RpoH_II_ regulon in *R. sphaeroides* [66]. Microsynteny analysis further revealed that *rpoE-chrR* is in close proximity to the photosynthetic gene cluster of *R. capsulatus* (*bch* and *puf* genes in Figure 4). This genetic arrangement cannot be found in closely related members within the *Rhodobacteraceae* (155 genomes analyzed in total), and it remains speculative whether this unique gene colocalization is purely coincidental or represents a strong functional relationship between photosynthesis and the response to ^1^O_2_.

### 3.4. Transcriptome Analysis of the Response to Singlet Oxygen in R. capsulatus

**Figure 5:**
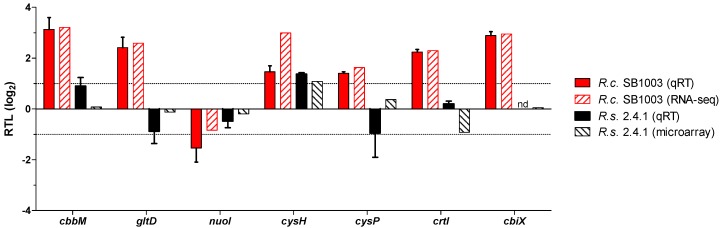
Gene expression changes for selected genes upon photooxidative stress in *Rhodobacter*. Relative transcript levels (RTL) were calculated after the onset of photooxidative stress in comparison to a non-stressed control in *R. capsulatus* (*R.c.* SB1003) and *R. sphaeroides* (*R.s.* 2.4.1). RNA-seq (this study) and microarray data [28] are shown for comparison. For qRT-PCR (qRT), bars represent the mean of biological triplicates, and error bars depict the standard deviation. The *cbiX* transcript was not detected (nd) in *R. sphaeroides*.

To learn more about the response to ^1^O_2_ in *R. capsulatus*, RNA-seq was performed for samples collected before (T0) and 10 min after the onset of ^1^O_2_ stress (T10). The reproducibility of biological replicates is shown by correlation analysis with highly significant Pearson’s *r*-values of ≥ 0.985 for all possible interreplicate comparisons (Figure S5). Principal component analysis (PCA) further revealed a clear separation between T0 and T10 samples along the first dimension (Figure S6A). In total, 3441 transcripts were quantified in the RNA-seq analysis. Four hundred and seventy-one transcripts were up- and 261 transcripts were down-regulated upon ^1^O_2_ stress (log_2_ fold change ≥ 1 or ≤ −1 and *p*-value < 0.05). To validate the RNA-seq approach for *R. capsulatus* and to further confirm the microarray results for *R. sphaeroides* [28], quantitative RT-PCR (qRT-PCR) was performed for selected genes. The qRT-PCR data were in good agreement with both RNA-seq and microarray results (Figure 5). However, for *cbbM*, *gltD*, *cysP*, *crtI*, and *cbiX*, transcript levels were only increased in *R. capsulatus*. In *R. sphaeroides*, three independent primer pairs were unable to detect *cbiX* transcripts.

**Figure 6:**
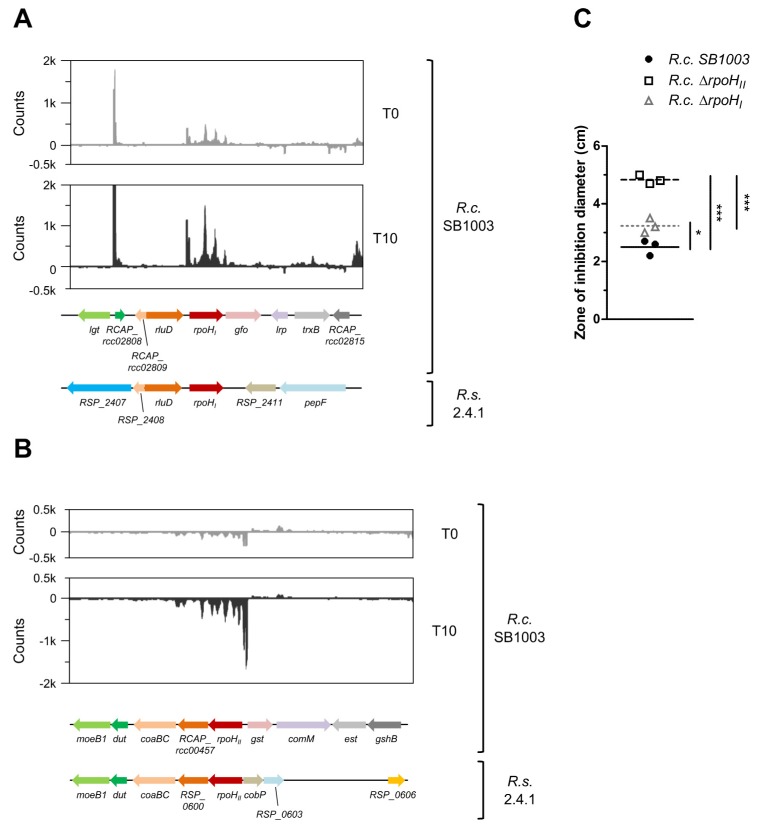
Analysis of *rpoH_I_* and *rpoH_II_* in *R. capsulatus*. RNA-seq and microsynteny analysis for (**A**) *rpoH_I_* and (**B**) *rpoH_II_* in *R. capsulatus*. The two upper panels show read count distributions of a representative RNA-seq experiment with *R. capsulatus* (*R.c.* SB1003) before (T0) and 10 min after the onset of photooxidative stress (T10). The lower panels show the microsynteny analysis of the *rpoH* locus in *R. capsulatus* and its homologous genes in *R. sphaeroides* (*R.s.* 2.4.1). Homologs are indicated by identical colors. (**C**) Zone of inhibition assay showing the sensitivity of *R. capsulatus* strains to photooxidative stress. Data points indicate individual measurements and bars represent the mean. One-way ANOVA followed by Bonferroni posttest was used to compare results from different *R. capsulatus* strains (* *p*-value < 0.05, *** *p*-value < 0.001).

From *R. sphaeroides*, it is known that 13 out of 15 genes of the RpoE regulon are induced by ^1^O_2_ (Table 1; [28]). In *R. capsulatus*, protein homologs were only found for seven of the 15 RpoE regulon members, which applies to RpoE, ChrR, photolyase PhrA (PhrB), sigma factor RpoH_II_, cytochrome c2 CycA (CycA1), a polyamine transporter subunit (PotH1), and cyclohydrolase FolE2 (RCAP_rcc01493) (Table 1). It is worth noting that the aforementioned *R. capsulatus* sigma factors RpoH_I_ and RpoH_II_ are wrongly annotated in public databases: our analyses (see below) clearly show that RCAP_rcc00458 (annotated as *rpoH_I_*) represents *rpoH_II_*, and vice versa, RCAP_rcc02811 (annotated as *rpoH_II_*) corresponds to *rpoH_I_* from *R. sphaeroides.* Thus, we refer to RCAP_rcc00458 as *rpoH_II_* and to RCAP_rcc02811 as *rpoH_I_*. Among the seven homologs of the RpoE regulon, only *rpoE*, *chrR*, *phrB*, *rpoH_II_*, and *folE2* were induced upon ^1^O_2_ stress in *R. capsulatus*, indicating that similarities are probably limited to the most important features, which has also been observed for *Roseobacter denitrificans* [67]. One of the conserved ^1^O_2_-related features includes sigma factor RpoH_II_, which shares a partially overlapping regulon with heat-shock sigma factor RpoH_I_ in *R. sphaeroides* [10,19,20]. In contrast to *rpoE-chrR*, the genetic context of both *rpoH_I_* and *rpoH_II_* is partly conserved between *R. sphaeroides* and *R. capsulatus* (Figure 6A,B). From *R. sphaeroides* it is known that RpoH_II_ is more important for ^1^O_2_ stress resistance than RpoH_I_ [20]; the same was observed here for *R. capsulatus* (Figure 6C).

**Figure 7:**
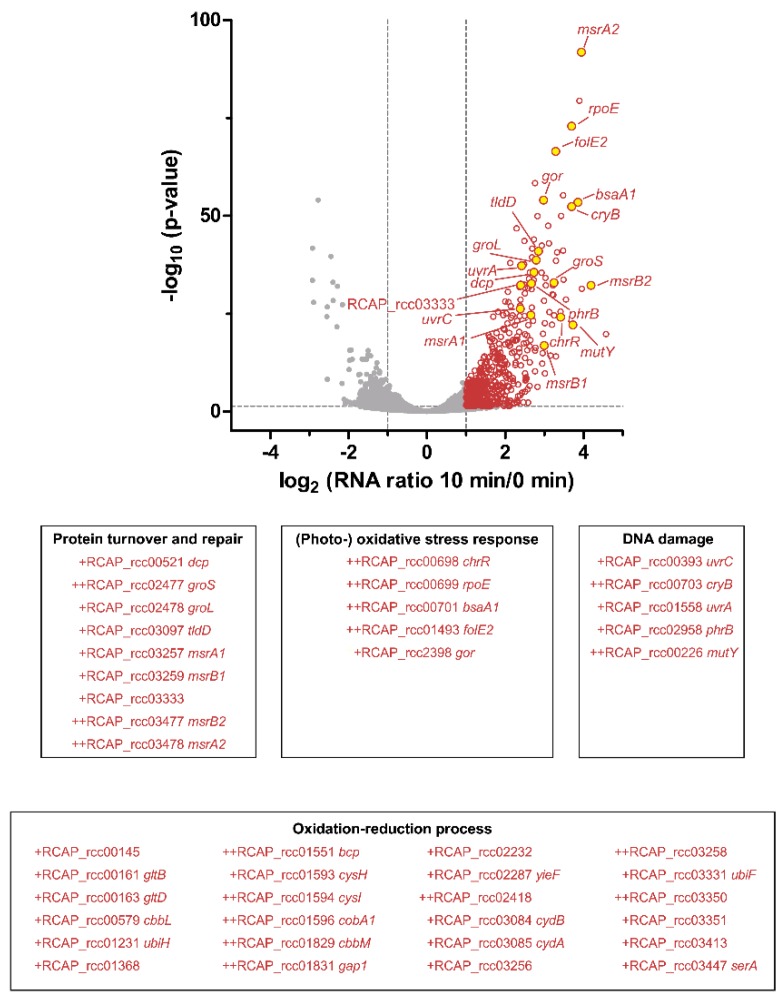
Transcriptome analysis of *R. capsulatus* reveals functional groups with importance to photooxidative stress. Changes in transcript abundance after 10 min of photooxidative stress were determined by RNA-seq of biological triplicates. The volcano plot depicts log_2_ fold changes (10 min versus 0 min) of all quantified transcripts and the corresponding *p*-values (as negative log_10_). The horizontal dashed line indicates the cutoff for statistical significance (*p* < 0.05), and the vertical dashed lines indicate log_2_ fold changes ≤ −1 and ≥ 1. Significantly increased transcripts with a log_2_ fold change ≥ 1 are indicated as red open circles. Transcripts encoding proteins with known stress-related functions are highlighted in yellow. Boxes below the volcano plot depict functional groups as determined by GO term enrichment analysis of the 100 transcripts with the strongest increase. Log_2_ fold changes of ≥ 2 or ≥ 3 are indicated with + and ++, respectively.

To identify the most prominently enriched functional groups in *R. capsulatus*, we conducted a Gene Ontology (GO) term enrichment analysis using BiNGO in Cytoscape [58,59] for the 100 transcripts with the strongest increase. Transcripts contributing to oxidation–reduction processes were significantly increased and formed the largest group (24 transcripts; Figure 7). Furthermore, we could identify several stress-related functional groups. The first group comprises nine transcripts encoding proteins with a role in protein turnover and repair, which applies to the peptide methionine sulfoxide reductases MsrA1, MsrA2, MsrB1, and MsrB2, the chaperones GroS and GroL, and the peptidases/proteases Dcp, TldD, and RCAP_rcc03333 (Figure 7). The second group comprises five transcripts encoding proteins with a known role in the (photo-) oxidative stress response, including RpoE and ChrR, RpoE-dependent GTP cyclohydrolase FolE2, peroxidase BsaA1, and glutathione-disulfide reductase Gor (Figure 7). The third and last group is formed by five transcripts encoding proteins with a function in DNA damage repair, which applies to photolyases PhrB and CryB, two components of the UvrABC complex, and the A/G-specific adenine glycosylase MutY (Figure 7).

### 3.5. Similar Proteins Fulfill Important Functions in Response to Singlet Oxygen in R. sphaeroides and R. capsulatus

**Figure 8:**
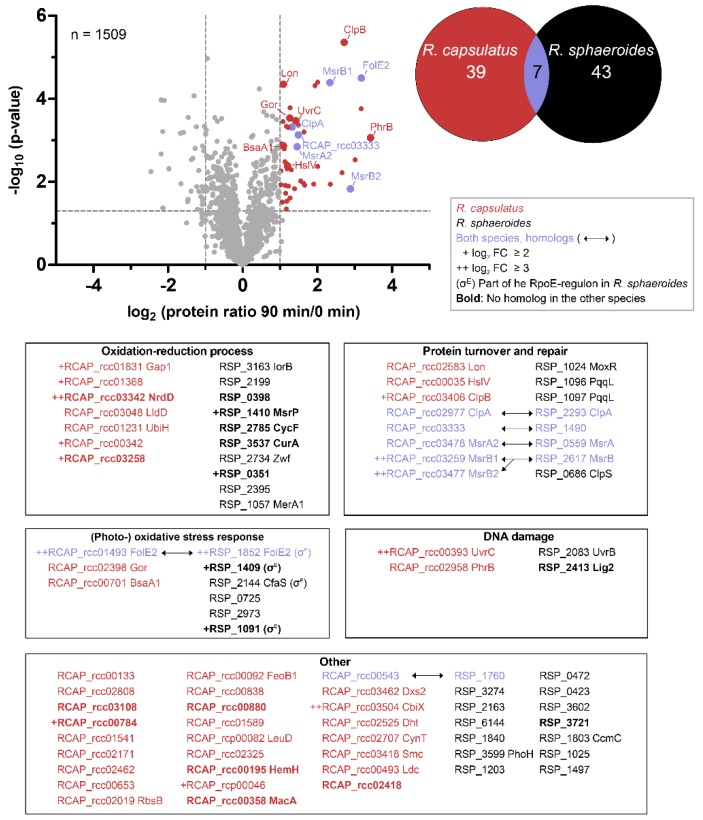
Functional characterization of proteins with increased abundance upon photooxidative stress. Changes in protein abundance after 90 min of photooxidative stress in *R. capsulatus* were determined by a label-free approach using LC-MS/MS of biological triplicates. The volcano plot depicts log_2_ fold changes (90 min versus 0 min) of all quantified proteins and the corresponding *p*-values (as negative log_10_). The horizontal dashed line indicates the cutoff for statistical significance (*p* < 0.05), and the vertical dashed lines indicate log_2_ fold changes ≤ −1 and ≥ 1. Significantly increased proteins with a log_2_ fold change ≥ 1 are highlighted. Proteome data were compared to *R. sphaeroides* [28] and increased proteins illustrated in an Euler diagram. Boxes below the volcano plot depict functional groups as determined by GO term enrichment analysis. Log_2_ fold changes of ≥ 2 or ≥ 3 are indicated with + and ++, respectively. Colors indicate whether proteins were found only in *R. capsulatus* (red), only in *R. sphaeroides* (black), or in both species (blue). See legend for details.

It is known that changes on the RNA level are not necessarily reflected by changes in protein abundance. We therefore complemented our dataset by proteomic analysis of samples collected before (T0) and 90 min after the onset of ^1^O_2_ stress (T90). Correlation between the transcriptome (T10) and proteome (T90) was fairly low (Pearson’s *r*-value = 0.49). Proteins were analyzed by LC-MS/MS and applied to a label-free quantification (LFQ) approach [68]. Pearson’s *r*-values of ≥ 0.975 for interreplicate comparisons demonstrated high reproducibility of the LFQ approach (Figure S7). Principal component analysis (PCA) further revealed a clear separation between T0 and T90 samples along the first dimension (Figure S6B). LFQ intensities, reflecting protein abundance, were subsequently used to calculate fold changes between conditions. In total, 1507 proteins were quantified, revealing 46 increased and 35 decreased proteins upon ^1^O_2_ stress (log_2_ fold change ≥ 1 or ≤ −1 and *p*-value < 0.05). Increased proteins were subjected to GO term enrichment analysis using BiNGO in Cytoscape [58,59], and compared to proteome data from *R. sphaeroides* [28]. Seven homologous proteins were identified as increased in both organisms (Figure 8), including the three methionine sulfoxide reductases MsrA, MsrB1, and MsrB2, GTP cyclohydrolase FolE2, ATP-dependent protease ClpA, a putative protease (RCAP_rcc03333/RSP_1490), and an uncharacterized protein (RCAP_rcc00543/RSP_1760). All other proteins were only found to be increased in one of the two organisms (39 proteins in *R. capsulatus* and 43 proteins in *R. sphaeroides*), and only one functional group was clearly enriched in both organisms by means of GO terms, i.e., proteins with a function in oxidation–reduction processes. However, we identified several proteins with stress-related functions and grouped them accordingly (Figure 8). A prominent group relates to protein turnover and repair, including the aforementioned methionine sulfoxide reductases and several proteases, that are either increased in both organisms (ClpA and RCAP_rcc03333/RSP_1490) or only in one of the organisms (Lon, HslV, and ClpB in *R. capsulatus*; PqqL, MoxR, and ClpS in *R. sphaeroides*). Other proteins are directly related to the (photo-) oxidative stress response, like glutathione-disulfide reductase Gor and glutathione peroxidase BsaA1 in *R. capsulatus*, and a thioredoxin (RSP_0725), a peroxiredoxin (RSP_2973), and several RpoE regulon members in *R. sphaeroides* (Figure 8). A last group includes proteins involved in DNA damage repair, like components of the UvrABC complex and photolyase PhrB in *R. capsulatus*. Regarding other noteworthy increases, the protein CbiX caught our interest. As the terminal enzyme of the siroheme biosynthesis [69], CbiX does not belong to any of the described groups; however, its increase was one of the strongest. As *R. sphaeroides* lacked a comparable increase of the CbiX-homolog, this could hint at a core difference in the photooxidative stress responses.

## 4. Discussion

A photosynthetic lifestyle allows organisms to use light as an energy source for growth and proliferation. However, this benefit comes at a price, that is, the risk of ^1^O_2_ generation by energy transfer from (bacterio-) chlorophyll to molecular oxygen (^3^O_2_) within photosynthetic complexes. It is suspected that purple bacteria from the genus *Rhodobacter* address this problem by avoiding strong pigmentation under high light and/or high oxygen conditions, a response that is mainly regulated by light- and oxygen-sensing proteins [2,3]. Despite regulation of pigmentation in response to oxygen tension and light, *Rhodobacter* species cannot completely avoid ^1^O_2_ stress in their natural aquatic environments. They have therefore evolved strategies to counteract ^1^O_2_ and to deal with the resulting damages [1,65]. *R. sphaeroides* is a well-studied model organism with regard to the photooxidative stress response [70], and was compared here to *R. capsulatus* using transcriptomics and proteomics. Intriguingly, the two *Rhodobacter* species elicit very similar responses in the light of functional categories. Many proteins with an increased abundance upon ^1^O_2_ exposure are involved in oxidation–reduction processes and DNA damage repair (Figure 8). Furthermore, several methionine sulfoxide reductases (MsrA and MsrB orthologs) and a variety of proteases were increased, which was also confirmed on the transcript level (Figure 7). Since proteins are the main targets of ^1^O_2_ [15], we conclude that the repair of proteins and the removal of damaged proteins is essential to survive this particular stress, and that efficient protein maintenance likely represents a key feature of the response to ^1^O_2_ in many organisms. In *R. sphaeroides*, the ^1^O_2_ stress response is mainly controlled by the alternative sigma factors RpoE and RpoH_II_ [10,18,19,20,21]; we have reason to believe that this also holds true for *R. capsulatus*. Firstly, both *rpoE* and *rpoH_II_* have elevated transcript levels upon ^1^O_2_ exposure (Table 1), and secondly, an *rpoH_II_* deletion strain is more sensitive to ^1^O_2_ than the *R. capsulatus* wild type (Figure 6). Hence, the basic regulatory principles of the photooxidative stress response might be very similar in both *Rhodobacter* species.

Despite the aforementioned similarities, there are remarkable differences between *R. sphaeroides* and *R. capsulatus*. Even though both species fall into the same clade within a phylogenetic tree based on a core genome of 580 orthologous proteins [71], synteny analysis revealed that the genetic organization of orthologous genes on the chromosome is considerably different, with the exception of, e.g., the photosynthetic gene cluster (Figure S8). This pronounced genomic rearrangement is not observed between different *R. sphaeroides* strains (e.g., strains 2.4.1, KD131, and WS8N), but between different genera within the *Rhodobacteraceae* (e.g., between *Rhodobacter* and *Roseobacter*). Hence, our analyses suggest that *R. capsulatus* and *R. sphaeroides* are, from a genomic point of view, more distantly related than expected from their common lifestyle and the arrangement of the photosynthetic gene cluster [71]. Another remarkable finding concerns the genetic context of the *rpoE-chrR* locus in *R. capsulatus*, which is in close proximity to the photosynthetic gene cluster (Figure 4). This genetic arrangement was not found in other *Rhodobacteraceae* species, and can therefore be considered as unique. It is worth noting that RpoE from *R. capsulatus* shares higher identity with the extra-cytoplasmic function sigma factor SigK from *Mycobacterium tuberculosis* (34%), compared to only 24% identity with *R. sphaeroides* RpoE. The same is true for *R. capsulatus* ChrR, which shares 25% identity with *M. tuberculosis* SigK antisigma factor RskA, but only 13% with *R. sphaeroides* ChrR. Phylogenetic trees further support the special position of the *R. capsulatus* RpoE and ChrR proteins within the *Rhodobacteraceae* family (data not shown). These findings raise the question of whether the *rpoE-chrR* locus in *R. capsulatus* and *R. sphaeroides* originated from a common ancestor and was then intensely remodeled in *R. capsulatus*, or whether *R. capsulatus* has received a *sigK-rskA*-like locus from another bacterial lineage (e.g., Gram-positives like *M. tuberculosis*) via horizontal gene transfer.

*R. capsulatus* not only displays a unique colocalization of the photosynthetic gene cluster near the *rpoE-chrR* locus; it also differs to *R. sphaeroides* on the physiological level with regard to its pigmentation. A higher pigmentation of *R. capsulatus* under high oxygen conditions obviously allows much faster adaptation to occur to phototrophic conditions compared to *R. sphaeroides* (Figure 1B). Despite this higher pigmentation, *R. capsulatus* has no disadvantage when exposed to sudden photooxidative stress (Figure 1C). Low expression of BChl *a* and carotenoid biosynthesis genes under aerobic conditions is reflected by low BChl *a* and carotenoid levels in both *R. capsulatus* and *R. sphaeroides* (Figure 2A). Upon photooxidative stress, Bchl *a* and carotenoid levels decreased in *R. sphaeroides*. By contrast, in *R. capsulatus,* only BChl *a* levels declined, while carotenoid levels remained fairly constant (Figure 2B), resulting in an increasing Crt:BChl *a* ratio in the course of ^1^O_2_ exposure (Figure 2D). These differences in Crt:BChl *a* ratio may be the reason for lower ^1^O_2_ levels in *R. capsulatus* under photooxodative stress considering the ^1^O_2_ quenching ability of carotenoids (Figure 3A). Increased carotenoid biosynthesis under conditions of photooxidative stress is a strategy that is used by some microorganisms, including the deltaproteobacterium *Myxococcus xanthus* and the yeast *Phaffia rhodozyma*, in order to avoid extensive cellular damages by direct quenching [72,73]. Accumulation of carotenoids upon ^1^O_2_ exposure was, however, not observed in *R. sphaeroides* [27], and omics data even revealed declining transcript and protein levels of genes from the photosynthetic gene cluster, including genes for carotenoid biosynthesis [28]. Interestingly, RNA-seq revealed that three genes within the photosynthetic gene cluster had increased transcript levels in *R. capsulatus*, which applies to *crtI* (log_2_ fold change of ~2.3), *crtB* (log_2_ fold change of ~2.0), and *tspO* (log_2_ fold change of ~1.3). Induction of *crtI* was validated by qRT-PCR (Figure 5). The three genes form an operon between the carotenoid biosynthesis genes *crtA* and *crtC*. TspO is an outer membrane protein, which controls efflux of porphyrin intermediates, and thereby negatively modulates expression of photosynthesis genes, likely through AppA [74]. The increased expression of *tspO* might enhance porphyrin efflux under photooxidative stress conditions, limiting both expression of photosynthesis genes and accumulation of potential porphyrin-derived photosensitizers, which may support adaptation to this particular stress. CrtB is a phytoene synthase and CrtI is a phytoene desaturase, which catalyze the reaction of precursors to phytoene (CrtB) and from phytoene to zeta-carotene and neurosporene (CrtI). The carotenoids spheroidene (SE) and spheroidenone (SO) are then synthesized in subsequent steps from neurosporene. Even though a switch from SE to SO (catalyzed by CrtA) might be an important adaption to photooxidative stress, we could not detect a strong increase for *crtA* mRNA in the RNA-seq data. It is conceivable that enhanced CrtB and CrtI levels are needed to provide sufficient amounts of neurosporene as a precursor for SE and SO biosynthesis to maintain carotenoid levels in *R. capsulatus* (Figure 2B). By contrast, in *R. sphaeroides crtI* and *crtB* transcript levels do not increase upon photooxidative stress (Figure 5) [28], which even coincides with a decrease in carotenoid levels (Figure 2B). Obviously, both *Rhodobacter* species use different strategies to adapt to high light regimes. Since *R. capsulatus* initially grows better than *R. sphaeroides* both after a shift to phototrophic conditions (Figure 1B) and upon photooxidative stress (Figure 1C), elevated carotenoid levels are expected to be advantageous to photosynthetic bacteria in rapidly changing environments.

Besides the protective function of carotenoids, *cbiX* might also play a more crucial role in defending against photooxidative stress in *R. capsulatus* than in *R. sphaeroides*. Our omics data show that *cbiX* is strongly increased on both transcript and protein level in *R. capsulatus* (log_2_ fold change of ~3). The RSP_1566 protein of *R. sphaeroides* shows 47% identity to CbiX, but neither protein nor transcript levels change significantly in response to ^1^O_2_ [28], and *cbiX* transcripts were not even detected by qRT-PCR (Figure 5). The *cbiX* gene is annotated as a cobaltochelatase which incorporates cobalt into sirohydrochlorin to form cosirohydrochlorin, an early precursor of vitamin B_12_ [75]. B_12_ has special functions in the formation of the photosynthetic apparatus in *Rhodobacter*, i.e., it is required for the conversion of protoporphyrin IX to Mg-protoporphyrin monomethyl ester [76]. Furthermore, it is needed by the antirepressor AerR to efficiently bind to repressor CrtJ, thereby inducing *bch* gene expression in *R. capsulatus* [77,78]. Theoretically, increased CbiX levels favor B_12_ biosynthesis and, consequently, derepression of *bch* genes via AerR. However, increased *bch* expression would then increase photooxidative stress, but this was not observed. A structural homology search for *R. capsulatus* CbiX using Phyre^2^ suggests that it might rather act as a ferrochelatase involved in the biosynthesis of siroheme. This assumption is supported by a study by Bali and colleagues [69], showing that CbiX functionally replaces characteristic siroheme biosynthesis enzymes, which are missing in alphaproteobacteria, including *R. sphaeroides*. As siroheme is a cofactor of nitrite and sulfite reductases, an increased siroheme production might also explain the high accumulation of the mRNA for sulfite reductase CysI (log_2_ fold change of ~3.2). Increased sulfur assimilation might help to counteract the ^1^O_2_-caused depletion of glutathione and abundant damages on sulfur-containing amino acids. More importantly, however, the insertion of Fe^2+^ into sirohydrochlorin reduces the concentration of free iron, which would otherwise be available for both the Fenton reaction and the formation of the photosensitizer protoporphyrin IX. Hence, additional stress is prevented by changing the flux through tetrapyrrole pathways [79,80,81]. As a conclusion, the induction of both the *crtIB-tspO* operon and *cbiX* might represent a successful strategy to respond to photooxidative stress, which was specifically invented in *R. capsulatus* to support adaptation. The question of whether other phototrophs have evolved similar strategies will be an exciting subject for future studies.

## Figures and Tables

**Table 1:** Transcriptome changes upon photooxidative stress of the RpoE regulon in *R. sphaeroides* compared to homologs in *R. capsulatus*.

*R. sphaeroides* Gene	Description	Log_2_ FC ^1 ^(7 min)	Log_2_ FC ^1 ^(45 min)	*R. capsulatus* Gene ^2^	Log_2_ FC ^1 ^(10 min)
RSP_1092 r*poE*	RNA polymerase sigma-70 factor	2.2 **	2.0 ***	RCAP_rcc00699 r*poE*	3.7 ***
RSP_1093 *chrR*	Antisigma factor ChrR	2.0 **	2.2 ***	RCAP_rcc00698 *chrR*	3.4 ***
RSP_2144 *cfaS*	Cyclopropane-fatty-acyl-phospholipid synthase CfaS	1.4 *	1.0 ***	RCAP_rcc00273 *rsmB1*	0.3
RSP_2143 *phrA*	DNA photolyase	1.6 **	1.5 ***	RCAP_rcc02958 *phrB*	2.7 ***
RSP_1091	Putative cyclopropane or cyclopropene fatty acid synthesis protein	2.2 ***	2.0 ***	No homolog	
RSP_1090	Putative cyclopropane/cyclopropene fatty acid synthesis protein	2.4	1.9 ***	No homolog	
RSP_1089	Sugar/cation symporter, GPH family	1.9 *	1.8 ***	No homolog	
RSP_1088	Hypothetical protein	1.1 *	0.8 **	No homolog	
RSP_1087	Short-chain dehydrogenase/reductase family member	0.9	0.7 **	No homolog	
RSP_0601 *rpoH_II_*	RNA polymerase sigma factor	2.0 *	2.1 *	RCAP_rcc00458 *rpoH_II_* ^3^	2.1 ***
RSP_1409	Beta-Ig-H3/fasciclin	2.8 **	4.5 *	No homolog	
RSP_1852 *folE2*	Hypothetical protein	2.2 ***	2.3 ***	RCAP_rcc01493 *folE2*	3.3 ***
RSP_0296 *cycA*	Cytochrome c2	−0.3	0.5 **	RCAP_rcc01240 *cycA1*	0.2
RSP_3336	ABC spermidine/putrescine transporter, inner membrane subunit	0.0	0.2 *	RCAP_rcc01895 *potH1*	−0.6 *
RSP_6222	Hypothetical protein	0.1	0.3	No homolog	

^1^ It is indicated whether log_2_ fold changes (log_2_ FC; stressed versus unstressed) were statistically significant (* *p*-value < 0.05, ** *p*-value < 0.01, and *** *p*-value < 0.001). Data for *R. sphaeroides* were retrieved from Berghoff and colleagues [28]. ^2^ Homologs found either by pBLAST (30% protein identity) or Phyre^2^. ^3^ Annotated as *rpoH_I_* in public databases.

## Supplementary Materials

The following are available online at https://www.mdpi.com/2076-2607/8/2/283/s1, Figure S1: Analysis of *Rhodobacter* transposon mutants (*R.c.* Tn5 and *R.s.* Tn5) lacking important carotenoids, Figure S2: Growth of *R. capsulatus* and *R. sphaeroides* with and without methylene blue in the dark or under high light conditions, Figure S3: Pigmentation of *R. capsulatus* and *R. sphaeroides* under microaerobic conditions, Figure S4: SOSG fluorescence in cell-free reactions, Figure S5: Correlation analysis of RNA-seq replicates, Figure S6: PCA of RNA-seq and LC-MS/MS replicate samples, Figure S7: Correlation analysis of LC-MS/MS replicates, Figure S8: Synteny plot comparing *Rhodobacter* genomes, Table S1: Strains used in this study, Table S2: Transcriptome data for *Rhodobacter* upon photooxidative stress, Table S3: Primers used for qRT-PCR in this study, Table S4: LC-MS/MS data for *R. capsulatus* upon photooxidative stress.
