# Supplementary material for: Adaptation to Photooxidative Stress: Common and Special Strategies of the Alphaproteobacteria Rhodobacter sphaeroides and Rhodobacter capsulatus
Source: Microorganisms. 2020 Feb 19;8(2):283. doi: 10.3390/microorganisms8020283 (PMC7074977; doi:10.3390/microorganisms8020283)
Supplement: Supplementary file 1 [file microorganisms-08-00283-s001.zip › Supplement/Supplementary Material.pdf]

microorganisms

MDPI

Supplementary Material

Adaptation to photooxidative stress: common and special strategies of the alphaproteobacteria *Rhodobacter sphaeroides* and *Rhodobacter capsulatus*

Mathieu K. Licht, Aaron M. Nuss, Marcel Volk, Anne Konzer, Michael Beckstette, Bork A. Berghoff and Gabriele Klug

This file contains:
Supplementary Figures S1–S8

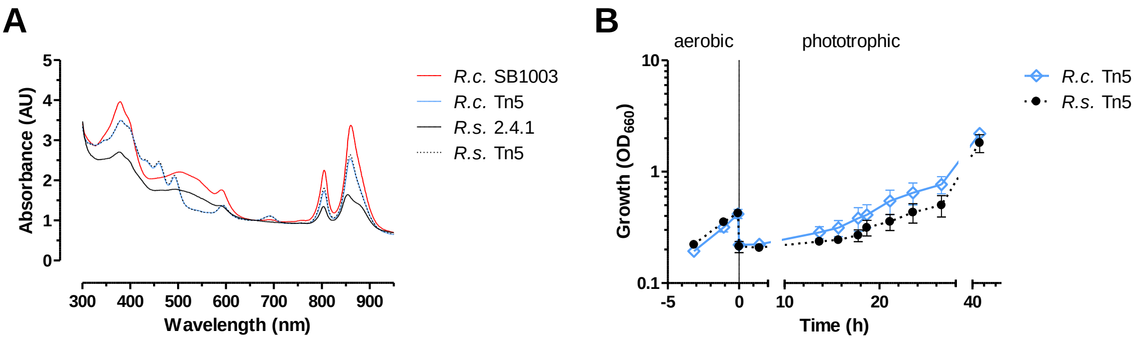

Figure S1. Analysis of *Rhodobacter* transposon mutants (*R.c.* Tn5 and *R.s.* Tn5) lacking important carotenoids. **(A)** Full-cell spectra indicate the lack of mature carotenoids, including spheroidene (SE) and spheroidenone (SO). **(B)** Growth of aerobically growing cultures shifted to phototrophic conditions. The OD_{660} was plotted semi-logarithmically against the time. Exponential phase cultures were diluted to an OD_{660} of 0.2 at time point 0 hours. Data points represent the mean of biological triplicates and error bars depict the standard deviation (standard deviations might not be visible if they are too small).

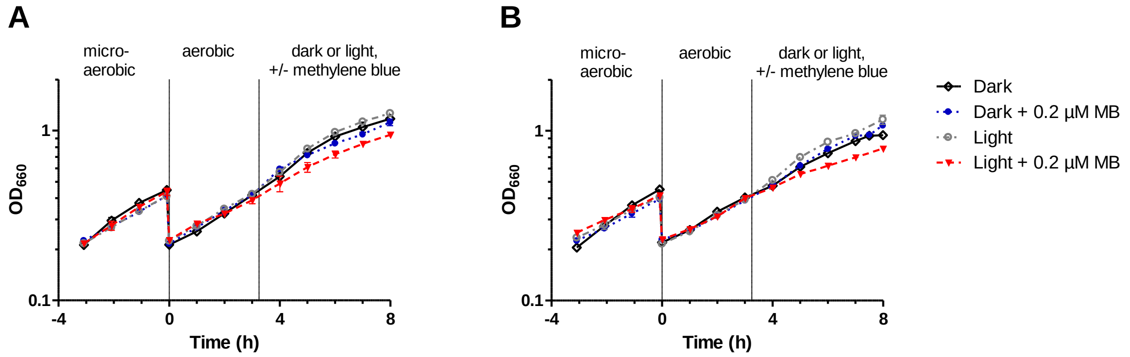

Figure S2. Growth of *R. capsulatus* **(A)** and *R. sphaeroides* **(B)** with and without methylene blue in the dark or under high light conditions. Cultures were gassed with air in flat glass bottles. Growth of biological triplicates of each species was monitored. Data points represent the mean of biological triplicates and error bars depict the standard deviation (standard deviations might not be visible if they are too small). Microaerobically growing cultures in the exponential phase were diluted to an OD_{660} of 0.2 at time point 0 hours and transferred to aerated bottles in the dark. Cultures were supplemented with methylene blue (final concentration 0.2 µM) or left untreated. Cultures were exposed to 800 W m^{-2} white light or kept in the dark at an OD_{660} of ~0.4.

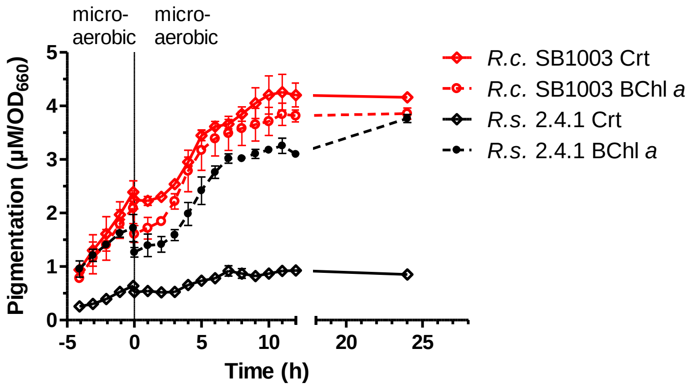

Figure S3. Pigmentation of *R. capsulatus* and *R. sphaeroides* under microaerobic conditions. Cultures in the exponential phase were diluted to an OD_{660} of 0.2 at time point 0 hours. The content of carotenoids (Crt) and bacteriochlorophyll *a* (Bchl *a*) were normalized to the respective OD_{660}-values and plotted against the time. Data points represent the mean of biological triplicates and error bars depict the standard deviation (standard deviations might not be visible if they are too small).

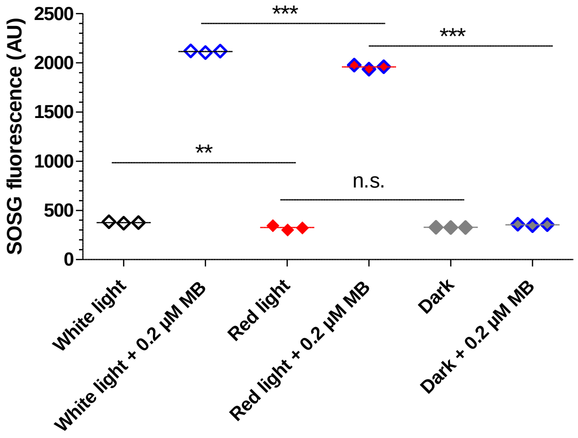

Figure S4. SOSG fluorescence in cell-free reactions. SOSG in HEPES-buffer (5 µM) incubated for 30 min at 32°C and 450 rpm in the presence of white light (800 W m^{-2}), red light (800 W m^{-2}) or in the dark with or without methylene blue (0.2 µM). Data points indicate individual measurements and bars represent the mean. Two-way ANOVA followed by Bonferroni post-test was used to assess results for selected comparisons (** p-value < 0.01, *** p-value < 0.001, n.s. not significant).

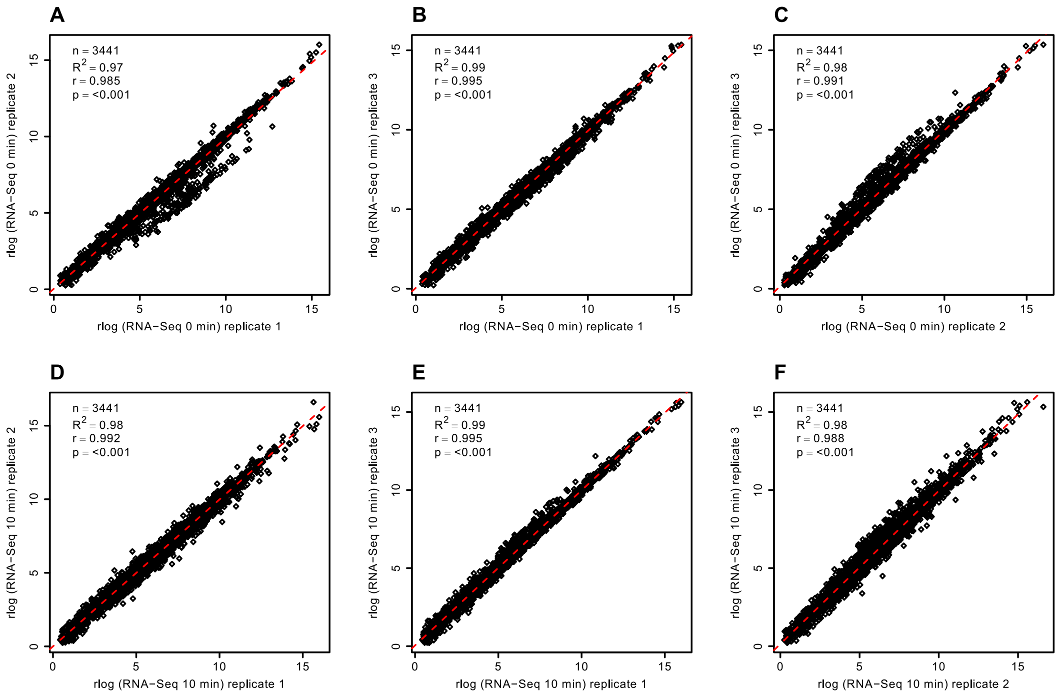

Figure S5. Correlation analysis of RNA-seq replicates. Read counts from RNA-seq analysis were normalized and rlog-transformed (DESeq2). Scatter plots show inter-replicate comparisons. A red dashed line indicates the linear regression. The number of transcripts (n), coefficient of determination (R^2), Pearson correlation (r) and p-value (p) are given.

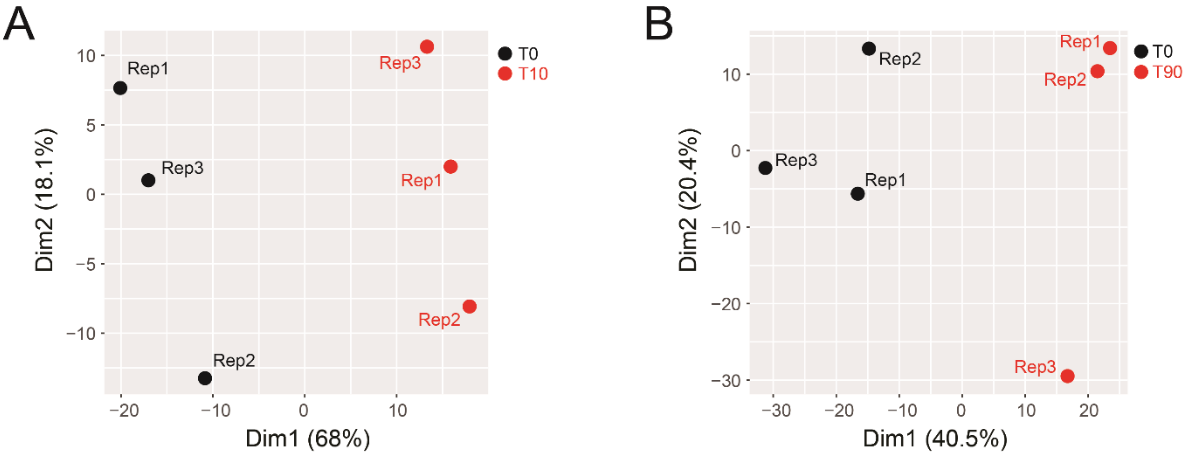

Figure S6. PCA of RNA-seq and LC-MS/MS replicate samples. Principal component analysis (PCA) of **(A)** rlog-transformed read counts from RNA-seq for 0 min (T0) and 10 min (T10) of photooxidative stress, and **(B)** LFQ intensities from LC-MS/MS for 0 min (T0) and 90 min (T90) of photooxidative stress in *R. capsulatus*. The position of individual replicates (Rep) within the first two dimensions (Dim) of the PCA is shown.

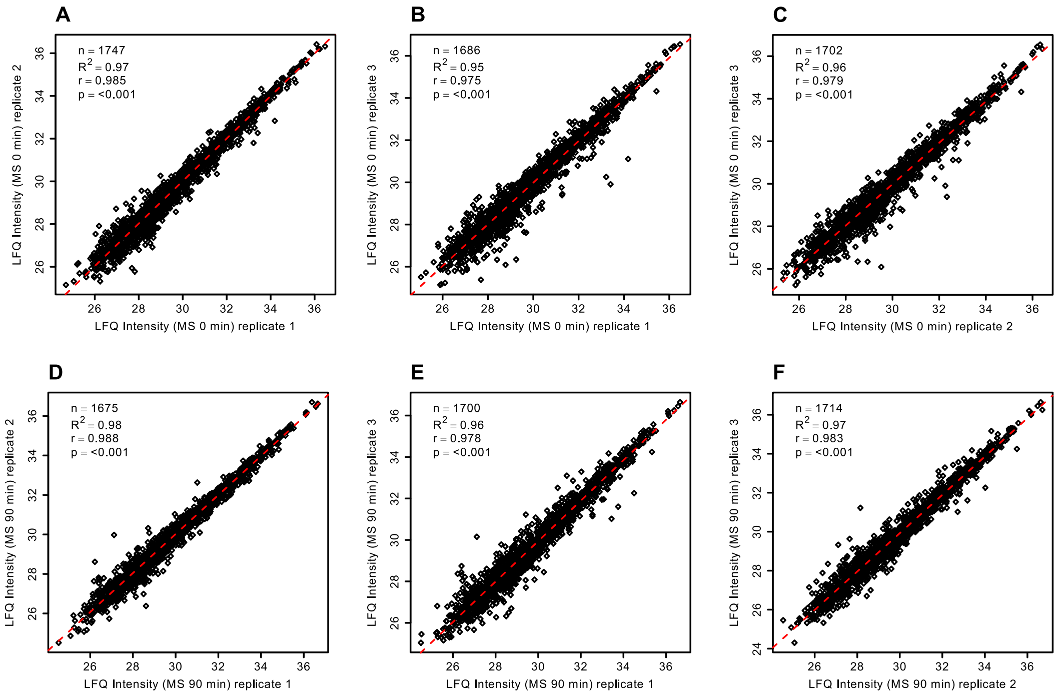

Figure S7. Correlation analysis of LC-MS/MS replicates. LFQ intensities were retrieved from LC-MS/MS analysis. Scatter plots show inter-replicate comparisons. A red dashed line indicates the linear regression. The number of transcripts (n), coefficient of determination (R^2), Pearson correlation (r) and p-value (p) are given.

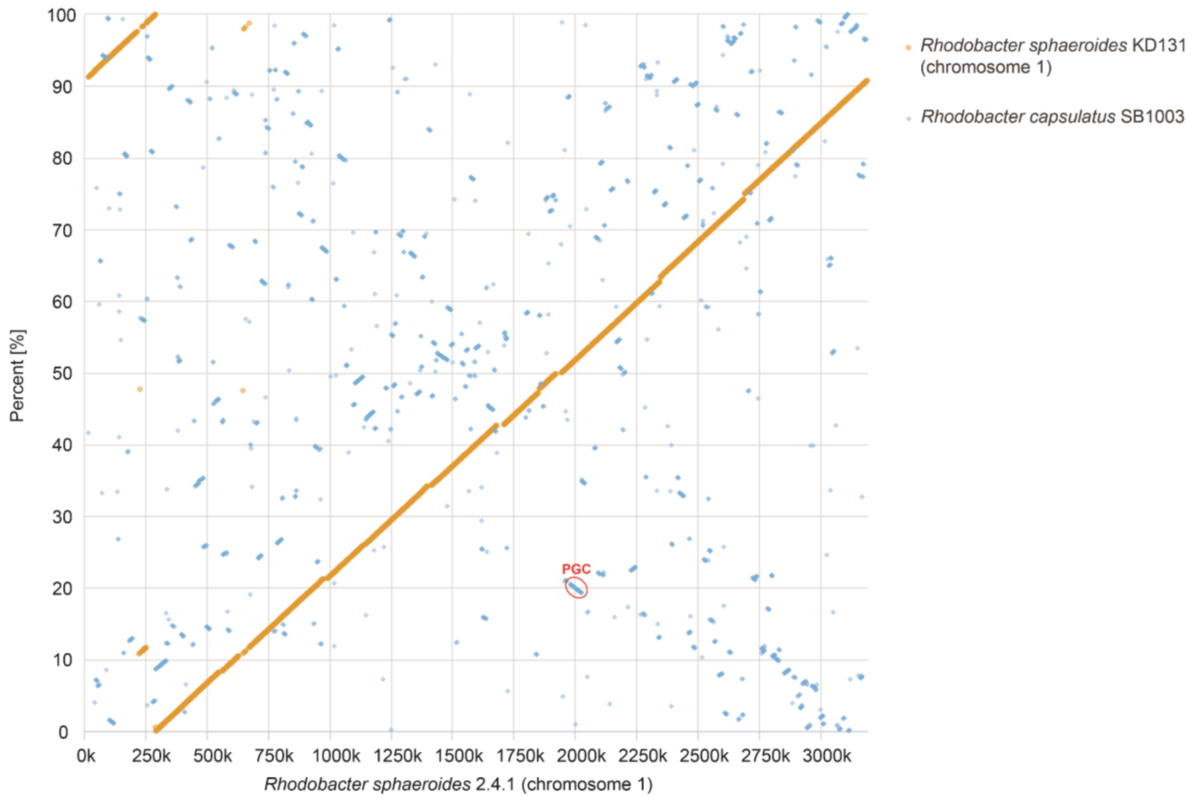

Figure S8. Synteny plot comparing *Rhodobacter* genomes. Chromosome 1 of *R. sphaeroides* 2.4.1 was used as reference and compared to chromosome 1 of *R. sphaeroides* KD131 (orange dots) and to the chromosome of *R. capsulatus* SB1003 (blue dots). The EDGAR 2.3 platform (https://edgar.computational.bio.uni-giessen.de) was used to generate a synteny plot for orthologous genes. The red circle indicates the position of the photosynthetic gene cluster (PGC).
