# Supplementary material for: Adaptation to Photooxidative Stress: Common and Special Strategies of the Alphaproteobacteria Rhodobacter sphaeroides and Rhodobacter capsulatus
Source: Microorganisms. 2020 Feb 19;8(2):283. doi: 10.3390/microorganisms8020283 (PMC7074977; doi:10.3390/microorganisms8020283)
Supplement: Supplementary file 1 [file microorganisms-08-00283-s001.zip › Supplement/Table S1.docx]

**Table S1.** Strains used in this study.

| **Strain** | **Description** | **Source** |
| --- | --- | --- |
| *Rhodobacter capsulatus* SB1003 | Wild type | [1] |
| *Rhodobacter sphaeroides* 2.4.1 | Wild type | [2] |
| *R. capsulatus* Δ*rpoH_I_* | SB1003 with disrupted *rpoH_I_*, Sp^R^ | [3] |
| *R. capsulatus* Δ*rpoH_II_* | SB1003 with disrupted *rpoH_II_*, Km^R^ | [3] |

Antibiotic resistances against spectinomycin (Sp^R^) and kanamycin (Km^R^) are indicated.

**References**

1. Yen, H.C.; Marrs, B. Map of genes for carotenoid and bacteriochlorophyll biosynthesis in *Rhodopseudomonas capsulata*. *J Bacteriol* **1976**, *126*, 619–629.

2. van Niel, C.B. The culture, general physiology, and classification of the non-sulfur purple and brown bacteria. *Bacteriol Rev* **1944**, *8*, 1–118.

3. Mercer, R.G.; Lang, A.S. Identification of a predicted partner-switching system that affects production of the gene transfer agent RcGTA and stationary phase viability in *Rhodobacter capsulatus*. *BMC Microbiol* **2014**, *14*, 71.
