# Supplementary material for: Adaptation to Photooxidative Stress: Common and Special Strategies of the Alphaproteobacteria Rhodobacter sphaeroides and Rhodobacter capsulatus
Source: Microorganisms. 2020 Feb 19;8(2):283. doi: 10.3390/microorganisms8020283 (PMC7074977; doi:10.3390/microorganisms8020283)
Supplement: Supplementary file 1 [file microorganisms-08-00283-s001.zip › Supplement/Table S3.docx]

**Table S3.** Primers used for qRT-PCR in this study.

| **Oligonucleotide** | **Sequence (5‘ → 3‘)** | **Target gene(s)** | **Efficiency** |
| --- | --- | --- | --- |
| rpoZ-for | GATGATCTGCGCGAGCGTCT | RCAP_rcc03318 (*rpoZ*) | 2.02 |
| rpoZ-rev | CCTTGCGCGTCCATCAATGC |  |  |
| 1669-A | ATCGCGGAAGAGACCCAGAG | RSP_1669 (*rpoZ*) | 2.02 |
| 1669-B | GAGCAGCGCCATCTGATCCT |  |  |
| RCC_01829_RTfor | GGCCTACATGCTGACCGA | RCAP_rcc01829 (*cbbM*) | 2.01 |
| RSP_3271_RTfor | GCGGCCGACAAGATCATG | RSP_3271 (*rbpL*) | 2.00 |
| RSP_3271_RTrev | CCCGAGGTCTGGATCACG | RCAP_01829 (*cbbM*)  RSP_3271 (*rbpL*) | 2.01 |
|  |  |  | 2.00 |
| RSP_1149_RTfor | CCCGCTGCACAACAACAT | RCAP_rcc00163 (*gltD*)  RSP_1149 (*gltD*) | 2.02  2.02 |
| RCC_00163_RTrev | CGCAGATTTCCGGGAAGC | RCAP_rcc00163 (*gltD*) | 2.02 |
| RSP_1149_RTrev | CTCGGGGAAGGTGTTGGT | RSP_1149 (*gltD*) | 2.02 |
| RCC_01531_RTfor | ATGAAAAGGGGCCGC | RCAP_rcc01531 (*nuoI*) | 1.95 |
| RCC_01531_RTrev | CAGATCGCCTCGCAAAGC |  |  |
| RSP_2523_RTfor | ACTACCCGCACGAGAAGG | RSP_2523 (*nuoI*) | 1.93 |
| RSP_2523_RTrev | TTCCGCGTCGATGGTGAT |  |  |
| RT-cysH-F | CGACGGCACGCTTTATCAGTT | RCAP_rcc01593 (*cysH*) | 1.97 |
| RT-cysH-R | ATTCTTCCAGATCCTCGCGC |  |  |
| 1941-A | TGCATCTCGTCTCGGTCATC | RSP_1941 (*cysH*) | 2.01 |
| 1941-B | TCGGTGTTGTAGCGGTTCAG |  |  |
| RT-cysP-F | GAAGGGGATCAAGGATTGGG | RCAP_rcc02744 (*cysP*) | 2.01 |
| RT-cysP-R | AGCGACAGATAGGCCTCGTT |  |  |
| 3697-A | AATGCGCGCTACACCTATCT | RSP_3697 (*cysP*) | 1.96 |
| 3697-B | GTCATGCACCCGGTAGAAAT |  |  |
| RCC_00679_RTfor | ACAAGGCCAAGGTGCTGA | RCAP_rcc00679 (*crtI*) | 2.06 |
| RCC_00679_RTrev | AAGCCCGTCCACCTCTTC |  |  |
| RSP_0271_RTfor | CCCTTCAACGTGACCTCGAT | RSP_0271 (*crtI*) | 2.05 |
| RSP_0271_RTrev | ACCTCGGTGTTCATGCGGAA |  |  |
| RT-cbiX-F | GCGCGGATCTATCCGTTCTT | RCAP_rcc03504 (*cbiX*) | 2.00 |
| RT-cbiX-R | CTCTGCGACCGTTTTCACCA |  |  |
